# Robust retrieval of biophysical traits under shade gradients in young *Hopea hainanensis* using visible and near-infrared hyperspectral hybrid modeling

**DOI:** 10.3389/fpls.2026.1788941

**Published:** 2026-06-18

**Authors:** Lin Chen, Xiaoli Yang, Xiaona Dong, Ling Lin, Mengmeng Shi, Feifei Chen, Chuanteng Huang, Huilin Yu, Ying Yuan, Miaoyi Han

**Affiliations:** 1 Hainan Academy of Forestry (Hainan Academy of Mangrove), Haikou, China; 2 The Innovation Platform for Academicians of Hainan Province, Haikou, China; 3 Research Institute of Forest Resource Information Techniques, Chinese Academy of Forestry, Beijing, China; 4 Precision Agriculture Lab, School of Life Sciences, Technical University of Munich, Freising, Germany

**Keywords:** canopy leaf dry mass, deep learning, *Hopea hainanensis*, hybrid, hyperspectral, scale normalization, shading, VNIR

## Abstract

**Introduction:**

Shading regulation is crucial for cultivating and restoring endangered *Hopea hainanensis* saplings. However, the high variability in light conditions introduced by shading gradients can increase noise in visible and near-infrared (VNIR) canopy hyperspectral reflectance and exacerbate the domain shift between measured spectra and those simulated by radiative transfer models (RTMs). In addition, dry leaf density (DLD) retrieval from VNIR spectra remains challenging. These limitations restrict the robust nondestructive monitoring of physiological and structural responses of saplings to shading.

**Methods:**

We developed an inversion framework that integrates multiple spectral scale-normalization methods with a hybrid RTM–deep learning modeling approach under controlled shading gradients. This framework was designed to improve cross-domain spectral consistency under shading-induced variability and to introduce a new carbon-based constituent index, DLD_CBC_, as a VNIR-compatible proxy for DLD.

**Results:**

Inter-group shading differences in original reflectance were easily obscured by noise, whereas scale normalization substantially enhanced the shading response in the green peak and red-edge regions and better-preserved physical consistency between RTM-simulated and field-measured data. The hybrid modeling achieved the highest accuracy for chlorophyll (R^2^ = 0.820; RMSE = 3.390 μg·cm^-2^), while nitrogen retrieval remained limited under VNIR (R^2^ = 0.404; RMSE = 0.207). DLD_CBC_ exhibited a consistent trend with measured DLD (r^2^ = 0.747; RMSE = 4.867 mg·cm^-2^). Both the inverted chlorophyll and DLD_CBC_ can effectively reproduce the directional response of measured traits along the shading gradient.

**Discussion:**

This study provides a practical VNIR-based pathway for nondestructive and robust retrieval of key biophysical traits of *H. hainanensis* saplings under highly variable shading conditions.

## 1 Introduction

*Hopea hainanensis* is a keystone tropical tree species of southern China and Southeast Asia (Song et al., 2020; Wang et al., 2021; Tang et al., 2024), listed as “Endangered” by the International Union for Conservation of Nature (IUCN) (Chen et al., 2020). Systematic research on the conservation, restoration, and sustainable utilization of *H. hainanensis* is of substantial ecological and economic importance (Luo et al., 2024; Yang et al., 2024). During population renewal and recovery, the seedling and sapling stages represent a decisive bottleneck for successful establishment and recruitment into forest stands, as physiological condition and functional traits directly determine survival and growth potential (Hirata et al., 2011; Saksa and Valkonen, 2011). Artificial cultivation is widely regarded as a fundamental approach to expanding *H. hainanensis* populations, alleviating pressure on wild resources, and enabling sustainable utilization (Wang and Meng, 2018; Yang et al., 2024; Huang et al., 2025). During its early growth stages, light environment regulation is one of the key factors influencing seedling survival and growth performance (Ji et al., 2020; Zhang et al., 2022). Shading treatments can significantly affect photosynthetic radiation acquisition (Walcroft et al., 2002; Huang et al., 2025), chlorophyll content (CHL) (Dai et al., 2009; Sonobe et al., 2018), nitrogen (N) allocation (Pons and Pearcy, 1994), and canopy structural traits (Cheng et al., 2022) and are therefore widely used in cultivation practice to mitigate excessive irradiance and water stress. However, the application of shading management requires careful balancing, as excessive shade may suppress photosynthetic capacity and induce morphological etiolation, while insufficient shade may fail to effectively alleviate environmental stress (Dai et al., 2009; Arellanos et al., 2025). At present, shading management for *H. hainanensis* saplings still relies largely on empirical judgment, lacking a quantitative understanding of how key biophysical traits respond to different shading intensities. This knowledge gap represents a major bottleneck for the precise cultivation and science−based management of this species.

Visible–near-infrared (VNIR) hyperspectral remote sensing has become a powerful, non-destructive approach for quantifying vegetation biophysical traits, yet retrieval performance is strongly trait dependent. Pigment-related traits such as chlorophyll content can be retrieved robustly from VNIR data because the green-peak and, in particular, the red-edge region are highly sensitive to pigment dynamics and consistently track their variation (Sonobe et al., 2018, 2020). Structural traits such as leaf area index (LAI) and biomass also tend to be retrieved reliably (Ningthoujam et al., 2025), benefiting from the strong sensitivity of near-infrared reflectance to canopy architecture. In contrast, VNIR-based retrieval of leaf mass per area (LMA) at the leaf scale and of its canopy-scale counterpart, dry leaf density (DLD; i.e., leaf dry matter per unit ground area), often carries substantial uncertainty. This limitation arises from two main factors: (i) LMA is jointly determined by multiple chemical and structural constituents whose VNIR spectral responses are highly covarying, making the signal difficult to disentangle and more prone to noise (Gara et al., 2021; Jin and Wang, 2022); (ii) Prior studies have consistently shown that reliable LMA retrieval typically requires shortwave infrared (SWIR) information, where diagnostically informative absorption features related to dry matter and proteins are present (Wang et al., 2011; Féret et al., 2019; Gara et al., 2021). Consequently, in many practical settings where only VNIR measurements are available, direct LMA retrieval is often inconsistent and unreliable (Yang et al., 2016). At the canopy scale, DLD, corresponding to LMA, is a key indicator of biomass accumulation. Because LMA is difficult to retrieve robustly from VNIR data alone, direct estimation of DLD is likewise challenging. This motivates the identification of VNIR-sensitive proxy variables that can indirectly characterize DLD variability under VNIR-only conditions.

To address the spectral complexity of LMA, the leaf radiative transfer model (RTM) PROSPECT and its extensions provide a key physical framework for understanding leaf spectral formation (Jacquemoud and Baret, 1990). The recently developed PROSPECT-PRO model decomposes LMA into a protein pool and carbon-based constituents (CBC), thereby parameterizing the distinct spectral contributions of these components and offering a new pathway for disentangling and retrieving leaf constituents (Féret et al., 2021). When coupled with the canopy radiative transfer model 4SAIL (Verhoef and Bach, 2007), the leaf-scale representation can be upscaled to the canopy level (PROSAIL-PRO), establishing a theoretical basis for multi-scale trait retrieval. Within this framework, the protein component is closely linked to leaf nitrogen, with diagnostically informative absorption features primarily located in the shortwave infrared (SWIR), whereas CBC represents a major fraction of leaf dry matter and more strongly reflects structural information (Féret et al., 2021). To date, most studies have emphasized the protein component in PROSPECT-PRO to improve nitrogen retrieval (Li et al., 2024; Ahmed et al., 2025), whereas CBC has received comparatively less attention. In particular, retrieving leaf-level CBC directly from canopy reflectance remains highly uncertain, largely due to scale mismatch between leaf parameters and canopy-scale observations. In this context, upscaling dry-matter information to a ground-area basis using LAI is physically more consistent with VNIR canopy hyperspectral measurements. Building on this rationale, we hypothesize that combining CBC with LAI to derive a CBC-based canopy proxy for dry leaf density (hereafter DLD_CBC_) can provide a more stable VNIR-sensitive indicator of canopy dry matter.

Moreover, in recent years, hybrid modeling that integrates physical mechanisms with data-driven approaches has become an important trend in hyperspectral trait retrieval. RTMs provide physics-based constraints on light–vegetation interactions, whereas deep learning models can efficiently capture non-linear relationships between spectral features and target traits (Cherif et al., 2023; Qi et al., 2026). Combining these approaches not only strengthens physical consistency and interpretability but also improves robustness and generalization under limited sample sizes (Berger et al., 2020; Bhadra et al., 2024; Zhang et al., 2026). However, when RTM-generated simulations are transferred to real observations, distributional discrepancies between simulated and measured data often arise, commonly referred to as domain shift (Zhang et al., 2025). In addition, under shaded gradient conditions, incident radiation intensity, shadow proportion, and within-canopy light distribution undergo significant changes. Highly variable environments introduce considerable spectral noise, which diminishes the consistency and comparability of spectral signals across different treatments (Seeley et al., 2023). This can markedly degrade the generalization of hybrid models across different shading conditions, and relying solely on model architecture optimization is unlikely to fully mitigate spectral inconsistencies driven by environmental variability. Previous studies suggest that standardization at the spectral scale levels can partially suppress noise effects and improve the comparability of spectral features across environmental conditions (Escribano et al., 2010; Kong et al., 2025). Nevertheless, how to systematically integrate multi-scale standardization strategies into an RTM–deep learning hybrid retrieval framework and quantitatively evaluate their effectiveness in high-variability, high-noise scenarios such as shading remains an underexplored research gap.

In summary, VNIR-based trait retrieval for *H. hainanensis* saplings under shading gradients remains challenging because shading increases spectral instability, while DLD is weakly represented in the VNIR region. This study aims to explore a feasible approach for the robust retrieval of key biophysical traits in *H. hainanensis* saplings under shaded gradient conditions using canopy VNIR hyperspectral data. The research focuses on CHL, leaf nitrogen content (N), and DLD as the primary retrieval targets. These traits were selected because they jointly describe key physiological, nutritional, and structural responses of saplings to shading. They also differ in their spectral sensitivity within the VNIR region. Therefore, their combined retrieval provides a multi-faceted assessment of sapling acclimation and growth status under shading management. To address these challenges, this study introduces a new CBC- and LAI-based canopy proxy for DLD under VNIR-only conditions and integrates spectral scale normalization with RTM–DL hybrid modeling to improve spectral comparability under shading-induced variability. Specifically, the study aims to: (i) address the limited capability of VNIR spectra to directly retrieve DLD by introducing CBC to construct a canopy-scale proxy for DLD, denoted DLD_CBC_, and to systematically evaluate its effectiveness as a stable VNIR-based surrogate for DLD across shading conditions; (ii) tackle the high environmental variability and potential spectral domain shift induced by shading gradients by developing multi-scale and spectral normalization strategies and integrating them into an RTM–DL hybrid retrieval framework to improve spectral comparability and model generalization across shading conditions; and (iii) on this basis, comprehensively assess the proposed framework in terms of retrieval accuracy for key traits and its capability to represent trait responses to shading. Overall, we try to establish a VNIR hyperspectral hybrid retrieval framework tailored to highly variable shading scenarios, with the goal of enabling robust, non-destructive estimation of biophysical traits in *H. hainanensis* saplings and providing methodological guidance for precision cultivation and shading management of endangered tree seedlings and saplings.

## 2 Materials and methods

### 2.1 Experimental design

**Figure 1:**
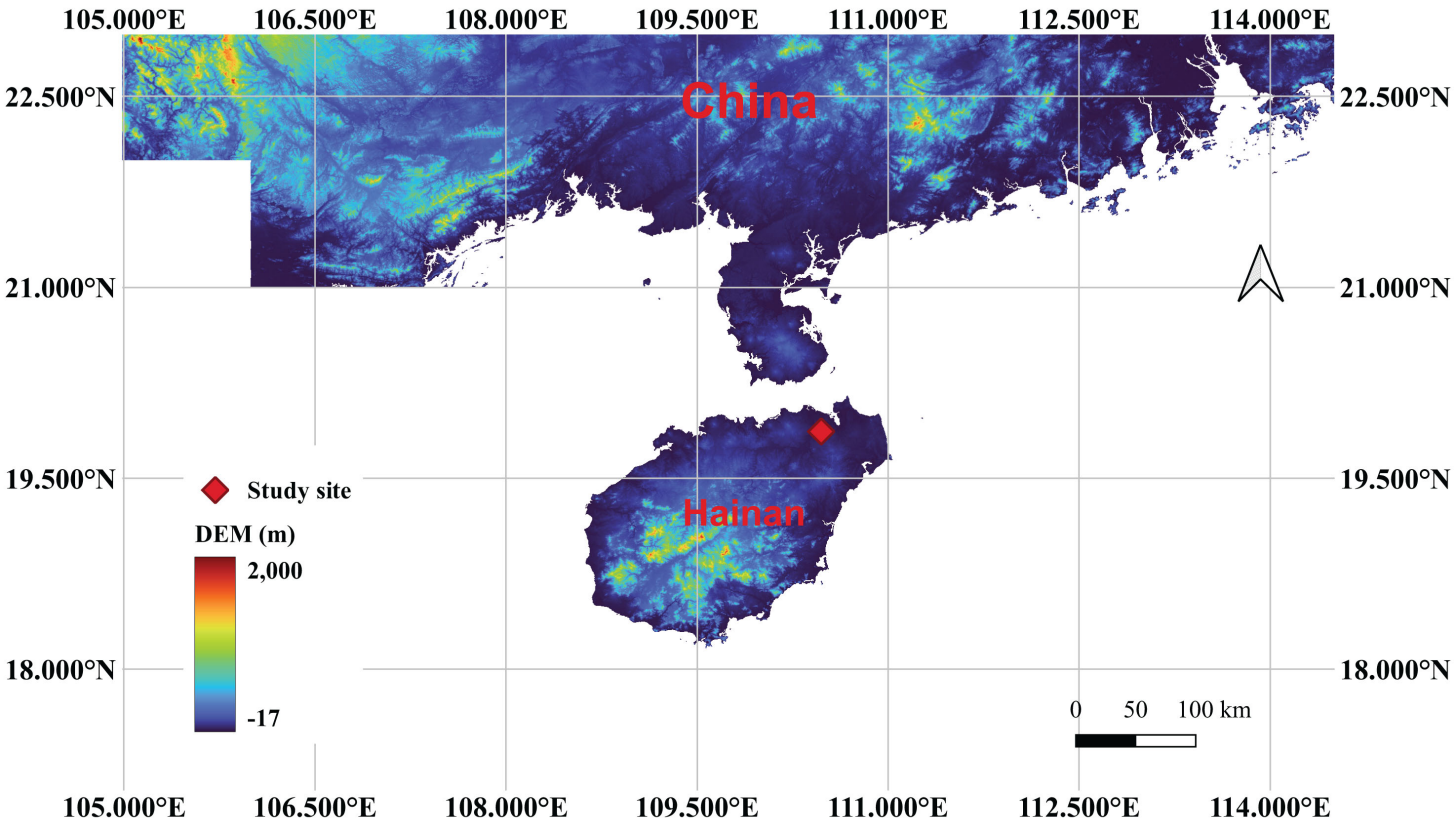
Geographic location of the controlled shading experiment for *Hopea hainanensis* saplings in Yunlong Town, Haikou City, China. The DEM background represents elevation, and the red diamond indicates the experimental site.

This study conducted a controlled shading experiment in Yunlong Town, Haikou City, China. The geographic location and elevation background of the experimental site are shown in **Figure 1**.

Shading gradients were established by installing black polyethylene shade nets (Lvandi, Greenland Shade Co., Taizhou, China) at a height of 2 meters above the ground. The experiment followed a completely randomized block design with four shading treatments, defined by shade intensity and light transmittance as follows: S1 (full sunlight control; 0% shading, 100% transmittance), S2 (light shading; 30% shading, 70% transmittance), S3 (moderate shading; 60% shading, 40% transmittance), and S4 (heavy shading; 90% shading, 10% transmittance). Subsequently, radiance measured from a calibrated white reference panel using a hyperspectral instrument was used to estimate incident irradiance and derive photosynthetically active radiation (PAR), enabling calibration of the effective transmittance for each treatment. The calibrated transmittance values were approximately 100%, 60%, 30%, and 10% for S1–S4, respectively. Each shading treatment included four replicate plots, with the control (S1) plot expanded to nine replicates to better capture the higher environmental variability under full-light conditions. All plots received uniform management, including weeding and irrigation.

**Figure 2:**
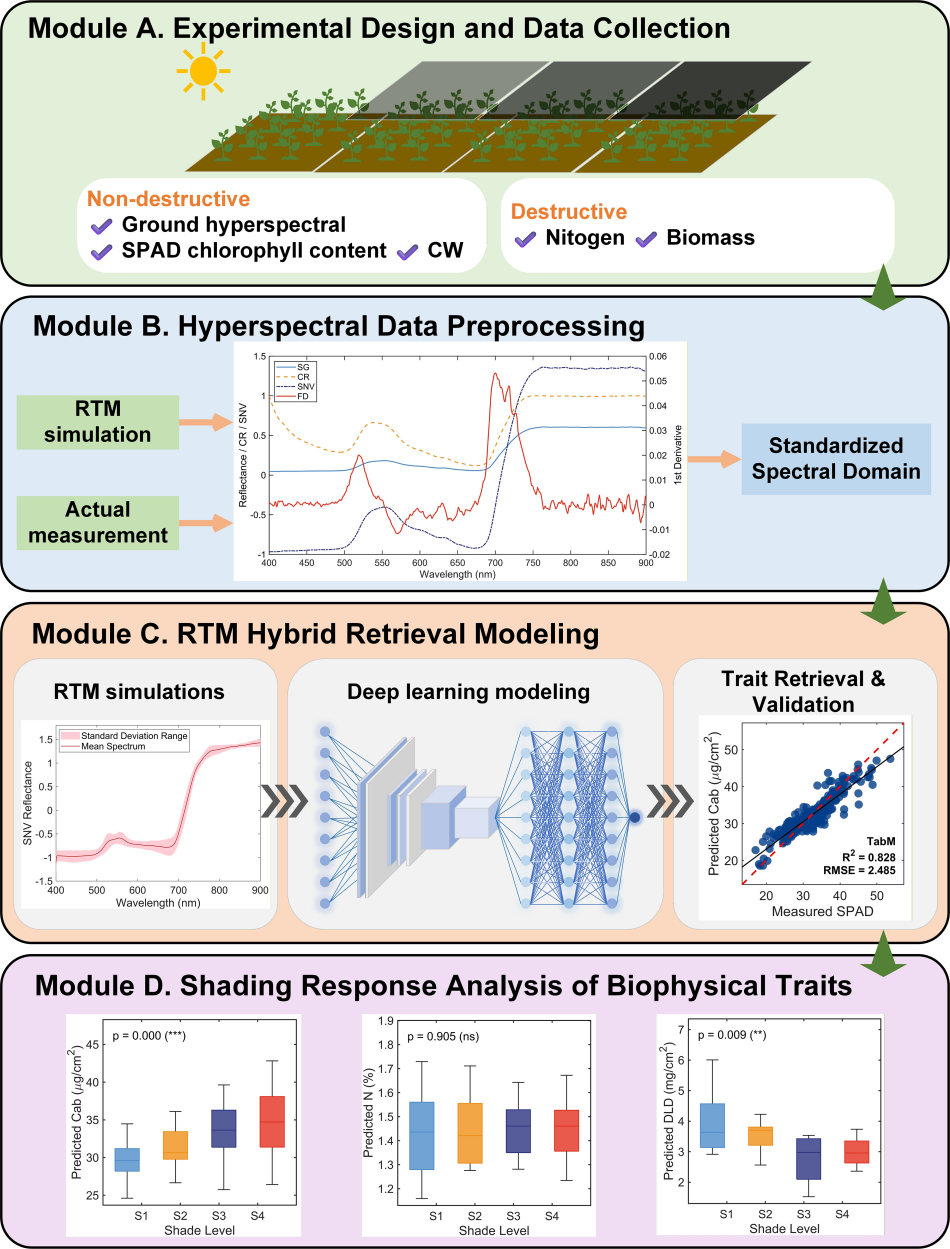
Workflow for retrieving biophysical traits of *H. hainanensis* under contrasting shading conditions using RTM and deep learning.

The overall workflow of this study is shown in **Figure 2**. It includes four main modules: experimental design and data collection, hyperspectral data preprocessing, RTM-based hybrid retrieval modeling, and analysis of shading-induced trait responses.

### 2.2 Data acquisition

#### 2.2.1 Hyperspectral data

Healthy *H. hainanensis* saplings were transplanted into the established shading experimental plots in June 2021 and were continuously grown under the assigned shading treatments throughout the experimental period. Ground-based canopy hyperspectral data were collected from the same batch of experimental saplings during two measurement campaigns, in November 2021 and June 2023. Given the need to balance the representativeness of healthy individuals with practical workload constraints, measurements were not conducted on all established saplings. Individuals exhibiting vigorous growth and no visible symptoms of pests or diseases were randomly selected from each plot as observation samples. In total, valid canopy hyperspectral data were obtained from 315 independent individual plants across both campaigns. A portable field spectroradiometer (PSR-1100f, Spectral Evolution, Lawrence, MA, USA) was used for measurements. Canopy hyperspectral data were collected under clear and cloud-free weather conditions to minimize fluctuations in ambient illumination. Because the experiment involved controlled shading treatments, before acquiring the canopy spectrum of each plant, a white reference panel measurement was performed for radiometric calibration to reduce the influence of short-term illumination variation and treatment-specific differences in incident radiation. The sensor probe was mounted vertically downward with a field of view of 25°. For each plant sample, the instrument automatically acquired ten consecutive scans within a few seconds, and the averaged spectrum was exported as the raw reflectance spectrum for subsequent analysis. Detailed instrument specifications are provided in **Table 1**.

**Table 1:** Instrument (PSR-1100f) specifications.

Instrument parameter	Specification
Spectral Range	325-1100nm
Spectral Resolution	3nm
Spectral Sampling Bandwidth	1nm
Noise Equivalence Radiance W/cm^2^/nm/sr	0.8x10^-9^
Wavelength Accuracy	± 0.5nm bandwidth

#### 2.2.2 Biophysical traits

Following the hyperspectral measurements, biophysical traits were assessed for the corresponding samples. Leaf chlorophyll status was quantified for all 315 samples using a portable chlorophyll meter, and each measurement was directly matched to the associated canopy hyperspectral observation. Since SPAD values indicate relative chlorophyll levels, calibration to absolute chlorophyll density (chlorophyll a+b; CHL, μg·cm^-2^) is typically required via empirical conversion. Numerous studies have demonstrated a robust, non-linear relationship between SPAD and CHL. Here, we adopted the calibration proposed by Coste et al (Coste et al., 2010). based on 13 tropical tree species (Equation 1), which has been reported to be broadly applicable across tropical taxa and has also shown strong performance in related studies on other plant species (Cerovic et al., 2012; Casa et al., 2015).

(1) $$CHL=\frac{117.1\times SPAD}{148.84-SPAD}\;\;\;\;R^{2}=0.89$$

To obtain key biochemical and structural parameters, representative subsamples were randomly selected from each shading treatment for destructive analysis. Fresh leaves were collected, oven-dried, ground, and analyzed for leaf N concentration using the Kjeldahl method, yielding 55 valid samples. In addition, plants were cut at ground level and separated into leaves and stems. Samples were oven-dried at 65 °C to constant mass and weighed to obtain leaf dry biomass (LDB), stem dry biomass (SDB), and total aboveground dry biomass (AGB). Crown projected area was estimated from the recorded east–west and north–south crown diameters, and leaf dry matter per unit area, defined here as dry leaf density (DLD; mg cm^-2^), was subsequently calculated, resulting in 40 valid samples. All destructive sampling was performed only after hyperspectral measurements had been completed, ensuring a fully paired “spectrum–trait” dataset.

### 2.3 Spectral data preprocessing

Since hyperspectral reflectance is sensitive to environmental noise and fluctuations in illumination, while shading treatments markedly alter the incident radiation received by the canopy, directly using raw reflectance spectra may amplify distributional discrepancies between radiative transfer model (RTM) simulations and field measurements. Such a mismatch can compromise the performance of subsequent hybrid modeling. To mitigate interference and improve model generalizability, we applied a standardized preprocessing pipeline to emphasize spectral shape and absorption features that are more directly linked to intrinsic plant biophysical traits.

To reduce high-frequency instrumental noise, Savitzky–Golay (SG) smoothing was first applied once to the raw spectra in the 400–900 nm range, using a window size of 11 and a third-order polynomial. Based on the SG-smoothed spectra, three complementary spectral representations were then generated separately: (i) continuum removal (CR) was performed to enhance and normalize absorption features; (ii) standard normal variate (SNV) transformation was applied to attenuate multiplicative effects associated with illumination intensity and scattering differences, thereby focusing the signal on shape-related variability; and (iii) first-derivative (FD) spectra were computed from the SNV-transformed spectra to further highlight wavelength-dependent rates. Through these steps, spectral data from different sources, including RTM-simulated spectra and field measurements under varying shading conditions, were mapped into a unified feature space focused on absorption characteristics and spectral shape, establishing a consistent foundation for subsequent hybrid model development.

### 2.4 Spectral feature selection

Although hyperspectral data contain abundant biophysical information, the numerous contiguous spectral bands can also introduce redundancy, leading to increased computational complexity and reduced model generalizability. To identify stable, trait-relevant spectral features, this study employed the Boruta all-relevant feature selection algorithm (Kursa et al., 2010). Boruta algorithm introduces randomly permuted “shadow features” as a statistical reference benchmark, thereby identifying true variables whose importance consistently surpasses that of random features across multiple iterations. The resulting subset of informative bands was used as input for subsequent model training and prediction.

### 2.5 Hybrid modeling framework

#### 2.5.1 PROSAIL-PRO radiative transfer model

In this study, we used the PROSAIL-PRO model, which couples the leaf-level PROSPECT-PRO model with the canopy-level 4SAIL model. By running the model forward with different parameter combinations, PROSAIL-PRO generates reflectance spectra from 400 to 2500 nm at 1-nm spectral resolution. Model input parameters were specified based on field measurements and values reported in the literature (**Table 2**) (Féret et al., 2021; Li et al., 2024). Using the parameter ranges listed in **Table 2**, 50,000 parameter combinations were generated using Latin hypercube sampling (LHS) to ensure broad and balanced coverage of the multidimensional parameter space. The resulting parameter combinations were then used as inputs for PROSAIL-PRO forward simulations to generate a synthetic spectrum–trait database for model training, including biophysical traits such as chlorophyll content and leaf area index (LAI), and simulated reflectance in the 400–900 nm range corresponding to the field hyperspectral measurements. Because the instrument used in this study has a spectral resolution of 3 nm with a 1-nm sampling interval, the simulated spectra were resampled to match the measured spectra using a Gaussian sensor spectral response function. The full width at half maximum (FWHM) of the Gaussian function was set to 3 nm according to the spectral resolution of the PSR-1100f (Schläpfer et al., 1999), and the resampled reflectance at each wavelength was calculated as the weighted average of the neighboring simulated reflectance values.

**Table 2:** Parameter settings for the PROSAIL-PRO model.

Model	Parameter	Unit	Range
PROSPECT-PRO	Leaf structure parameter (*N_struc_*)	–	1-2
	Leaf chlorophyll content (*C_ab_*)	μg·cm^−2^	10-60
	Leaf water content (EWT)	cm	0.001-0.05
	Leaf carotenoid content (*C_cx_*)	μg·cm^−2^	0-20
	Leaf anthocyanin content (*C_anth_*)	μg·cm^−2^	0.5-5
	Leaf protein content (*C_p_*)	mg·cm^−2^	0-3
	Carbon-based constituents (CBC)	mg·cm^−2^	0-10
4SAIL	Leaf area index (LAI)	m^2^·m^−2^	0-4
	Leaf inclination distribution function (LADF)	–	1, 2, 3, 4
	Sun zenith angle (SZA)	°	0, 30, 45
	Observer zenith angle (OZA)	°	0

LADF type: 1 = planophile; 2 = erectophile; 3 = plagiophile; 4 = spherical.

In the PROSAIL-PRO model, the parameter (*C*_p_) is closely related to leaf nitrogen and is therefore commonly used as a proxy for leaf N. Based on the protein–nitrogen conversion model, leaf nitrogen on an area basis is calculated using Equation 2:

(2) $$N_{area}=\frac{C_{p}}{k_{p}}$$

where (*k_p_*) is the nitrogen-to-protein conversion factor and was set to 4.43 in this study based on previous research (Féret et al., 2021; Li et al., 2024; Yeoh and Wee, 1994).

We converted the above area-based nitrogen to mass-based nitrogen using Equation 3 to match the field measurements:

(3) $$N_{mass}=\frac{C_{p}}{(C_{p}+CBC)\times k_{p}}\times 100\%$$

Additionally, the CBC parameter in the PROSAIL-PRO model is closely related to leaf dry matter. In this study, CBC was combined with LAI to generate a new canopy structural index DLD_CBC_. Theoretically, this derived index DLD_CBC_ and the field-measured DLD are directly related, as both represent leaf dry mass per unit ground area.

(4) $$DLD\propto DLD_{CBC}=CBC\times LAI$$

#### 2.5.2 Deep learning algorithms

Deep learning constitutes a core component of the hybrid retrieval framework, as it enables modeling of the complex, non-linear relationships between hyperspectral signatures and biophysical traits of *H. hainanensis*. We evaluated three deep learning models: deep neural network (DNN), residual network (ResNet), and TabM.

The DNN is a classical multilayer perceptron composed of stacked fully connected layers through successive non-linear transformations (Hinton et al., 2006). It extracts high-dimensional spectral representations and captures the global mapping between spectral features and target traits. ResNet extends deep architectures by introducing residual connections (He et al., 2015), which alleviate vanishing gradients and help preserve informative local spectral details. TabM is an enhanced architecture tailored for tabular data by emulating an ensemble of multilayer perceptrons (Gorishniy et al., 2025). It features parallel training and weight sharing, substantially improving computational efficiency and the capacity to fit tabular inputs.

#### 2.5.3 Model development and evaluation

Using the simulated “spectrum–trait” database of 50,000 samples generated from forward runs of PROSAIL-PRO, we split the dataset into 80% for training and 20% for validation to develop baseline spectral–trait models with DNN, ResNet, and TabM. The DNN consisted of a fully connected feature extractor with hidden dimensions of 512, 256, and 128, followed by a 64-unit regression layer. The ResNet contained two residual blocks with 256 hidden units. The TabM model was implemented as a parameter-efficient ensemble with 32 subnetworks, three MLP blocks, and a hidden dimension of 512. All models used ReLU activation, dropout regularization, MSE loss, and early stopping. The models were optimized using AdamW. The field-measured canopy hyperspectral data of *H. hainanensis* were then used as an independent test set to retrieve key biophysical traits, including CHL, N, CBC, and LAI, and retrievals were evaluated against the corresponding ground measurements. Model performance was assessed using the coefficient of determination (R²), root mean square error (RMSE), and squared Pearson correlation coefficient (r²), as defined in Equations 5–7:

(5) $$R^{2}=1-\frac{\sum_{i=1}^{n}{\left(y_{i}-{\hat{y}}_{i}\right)}^{2}}{\sum_{i=1}^{n}{\left(y_{i}-\bar{y}\right)}^{2}}$$

(6) $$RMSE=\sqrt{\frac{1}{n}\sum_{i=1}^{n}{\left(y_{i}-{\hat{y}}_{i}\right)}^{2}}$$

(7) $$r^{2}={\left[\frac{\sum_{i=1}^{n}(y_{i}-\bar{y})\left({\hat{y}}_{i}-\bar{\hat{y}}\right)}{\sqrt{\sum_{i=1}^{n}{\left(y_{i}-\bar{y}\right)}^{2}\sum_{i=1}^{n}{\left({\hat{y}}_{i}-\bar{\hat{y}}\right)}^{2}}}\right]}^{2}$$

Here, R^2^ quantifies the goodness of model fit, RMSE reflects the magnitude of retrieval errors, and r^2^ characterizes the similarity in linear trends between retrieved and measured values.

## 3 Results

### 3.1 Spectral responses to shading gradients

**Figure 3:**
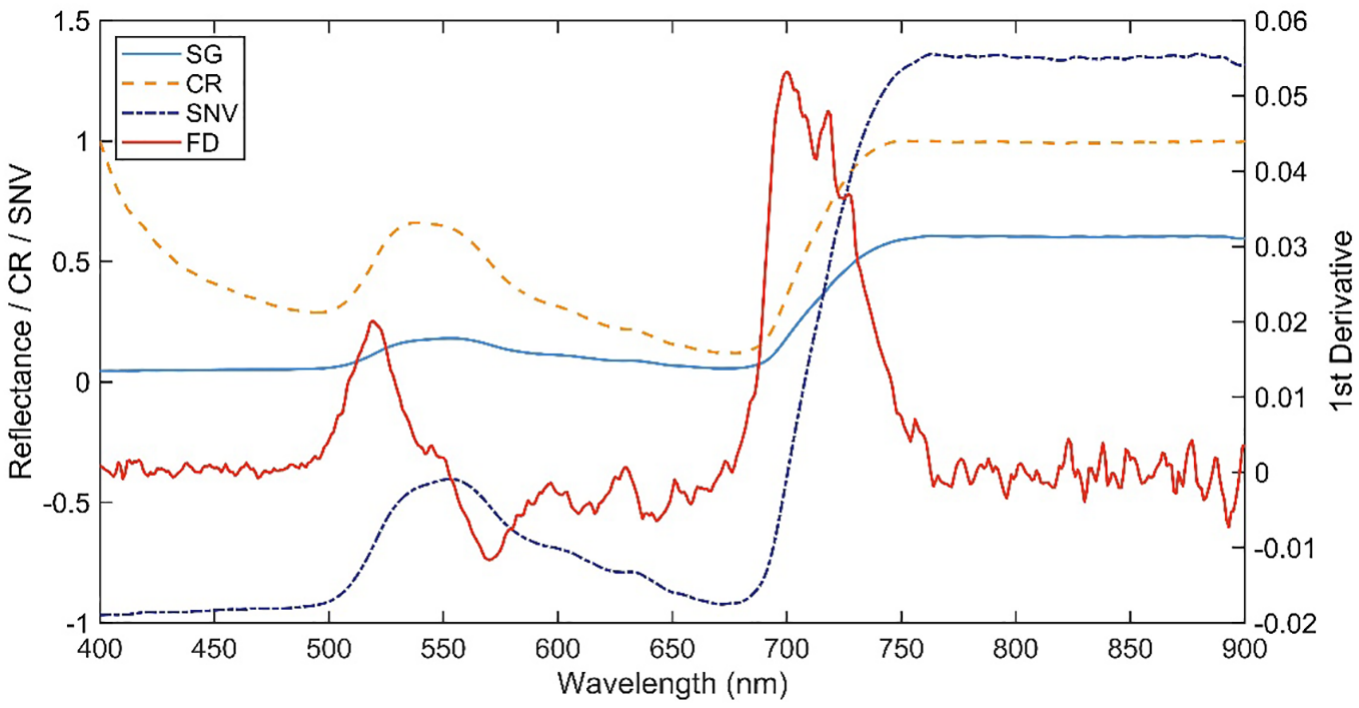
Comparison of four spectral preprocessing methods.

**Figure 3** shows representative examples of the spectral smoothing and transformation procedures. SG smoothing reduced high-frequency fluctuations in the original spectra, whereas CR, SNV, and FD emphasized different aspects of spectral information. CR enhanced absorption-related features, SNV reduced amplitude differences associated with illumination and scattering, and FD highlighted local changes in spectral slope. These complementary transformations provided the basis for evaluating whether spectral normalization could improve the separability of shading treatments and the consistency between RTM-simulated and field-measured spectra.

To quantitatively evaluate the response stability and inter-group separability under shading conditions across different spectral transformations, we calculated the Fisher Discriminant Ratio (FDR) for the full spectrum as well as for key spectral regions (the green peak and the red edge). Lower FDR values indicate that between-group differences are weak relative to within-group variability (noise). As shown in **Table 3**, the original reflectance exhibited low inter-group separability across all spectral ranges, with an FDR of only 0.130 in the green peak region, suggesting that differences among shade treatment groups were obscured by noise. In contrast, CR, SNV transformation, and FD processing increased FDR to varying degrees, and all yielded FDR values > 1 in the red-edge region, demonstrating markedly improved discrimination among shading treatments after spectral standardization.

**Table 3:** Evaluation of shading level separability (FDR) across various spectral transformations and waveband regions.

Spectral transformation	Full spectrum (400–900 nm)	Green peak (500–600 nm)	Red edge (680–760 nm)
Original	0.393	0.130	0.331
CR	0.444	0.799	1.269
SNV	0.950	1.032	1.089
FD	0.355	0.556	1.075

**Figure 4:**
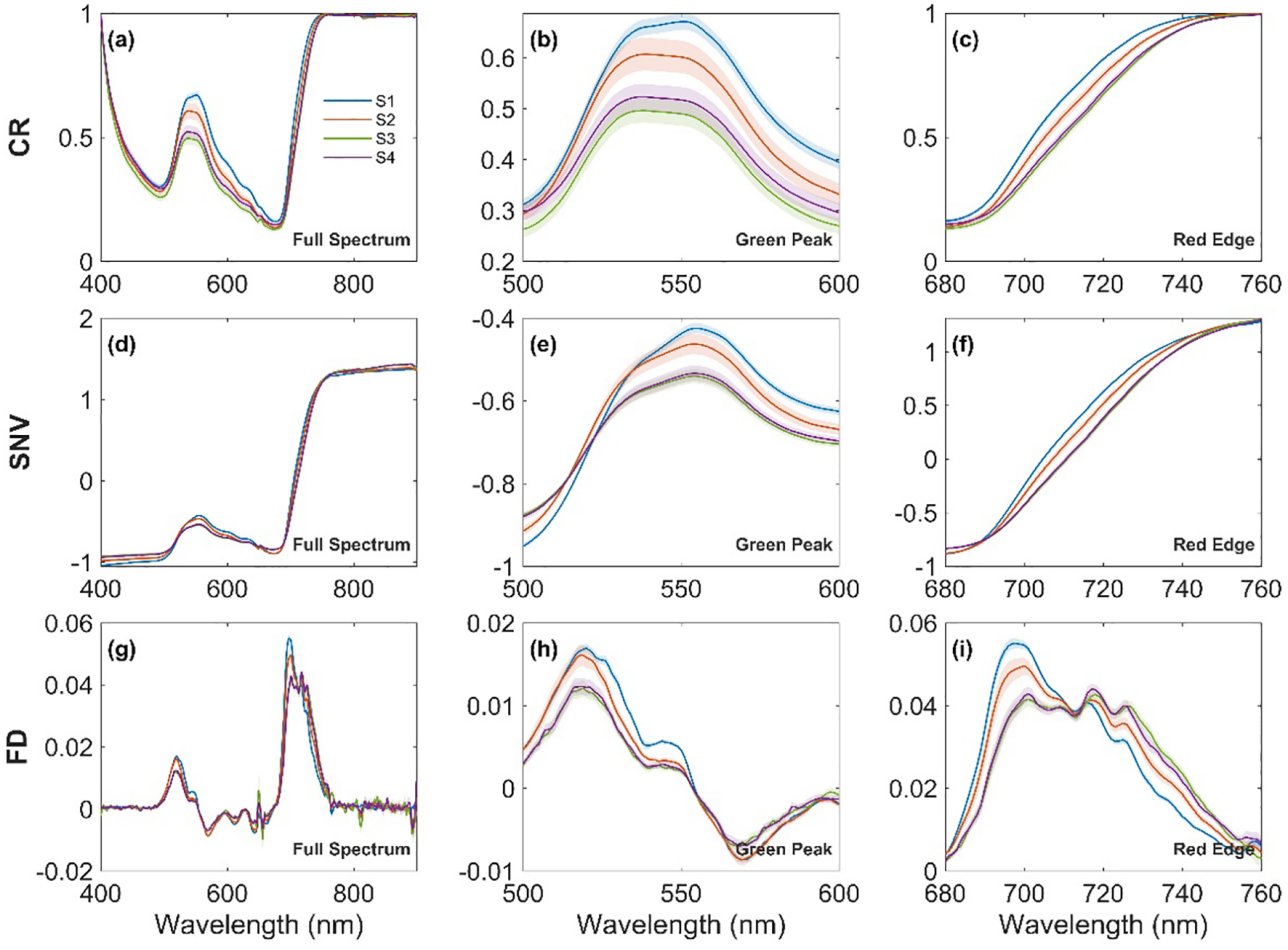
Variation in CR, SNV, and FD spectra across the full spectral range, green-peak region (500–600 nm), and red-edge region (680–760 nm) under different shading treatments. Panels **(a–c)**, **(d–f)**, and **(g–i)** show the CR, SNV, and FD spectra, respectively. Within each row, the panels represent the full spectral range, green-peak region, and red-edge region, respectively. S1 to S4 represent full sunlight, light, moderate, and heavy shading, respectively.

Furthermore, the canopy hyperspectral data (400–900 nm) after CR, SNV transformation, and FD processing were grouped by shading level (S1–S4), and group-mean spectra were calculated (**Figure 4**). From the full−range spectral curves (**Figures 4a, d, g**), differences among shading treatments were mainly observed in the green−peak and red−edge regions. In the green-peak region (500–600 nm; **Figures 4b, e, h**), the CR-, SNV-, and FD-processed spectra consistently decreased with increasing shading intensity. Notably, CR spectra still exhibited a clear separation between moderate shading (S3) and heavy shading (S4), whereas the SNV and FD spectra of S3 and S4 were highly similar, indicating reduced discrimination at higher shading levels. In the red-edge region (680–760 nm; **Figures 4c, f, i**), both CR and SNV spectra showed an overall decline with increasing shading. For the FD spectra, values decreased with increasing shading before the red-edge inflection point (REIP; ~715–720 nm), but exhibited the opposite pattern after the REIP, increasing with increasing shading. Consistent with the green peak results, S3 and S4 remained very close in their CR, SNV, and FD spectral signatures, suggesting comparable red-edge responses under moderate to heavy shading.

### 3.2 Consistency of spectral–trait relationships between RTM simulations and field measurements

**Figure 5:**
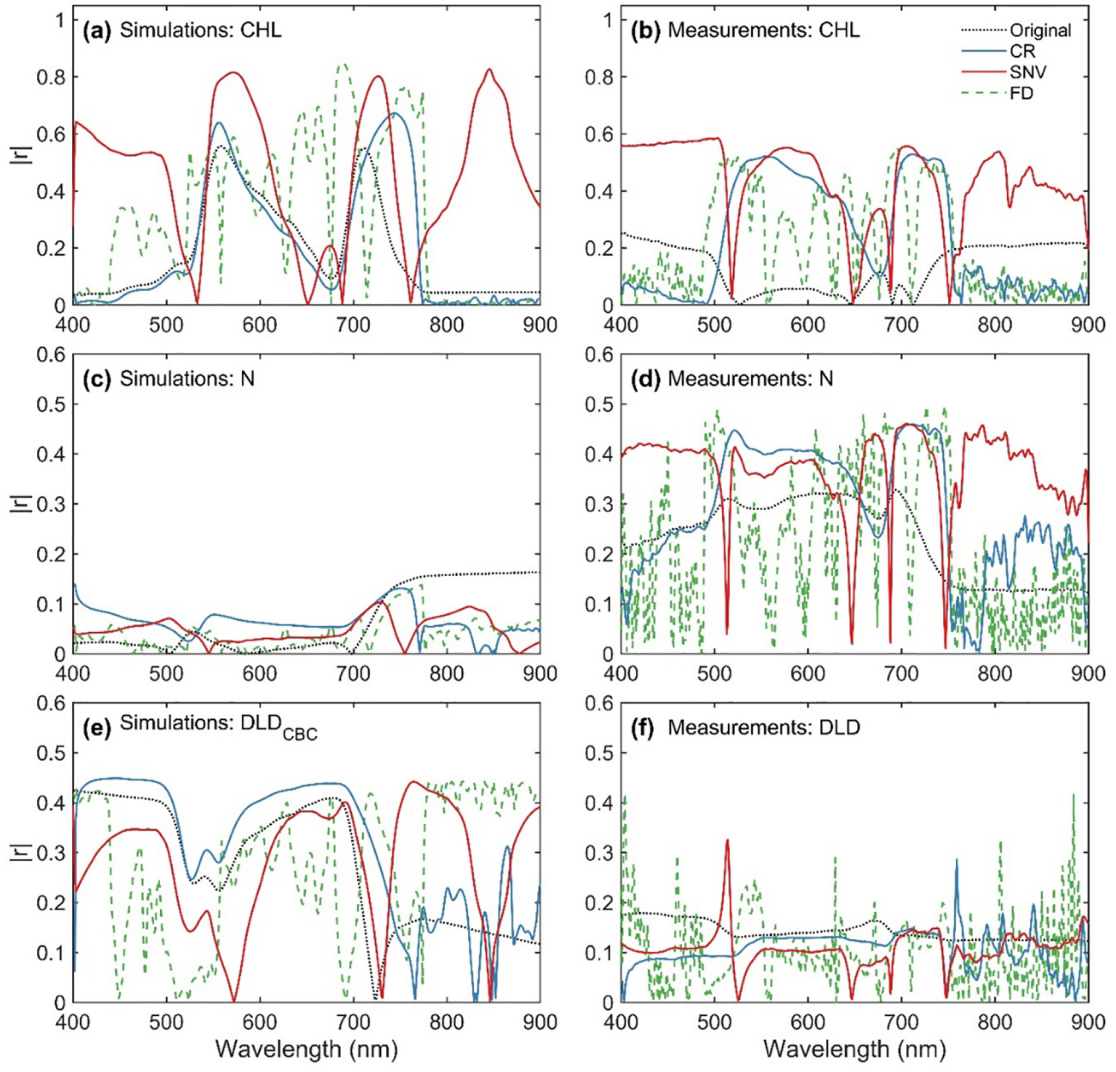
Comparison of spectral–trait correlations for Original, CR-, SNV-, and FD-transformed spectra between simulated and field-measured datasets. Panels **(a, c, e)** show the simulated relationships for CHL, N, and DLD_CBC, respectively, whereas panels **(b, d, f)** show the corresponding field-measured relationships for CHL, N, and DLD, respectively.

For the PROSAIL-PRO–simulated LUT dataset and the field-based paired spectral–trait dataset, we computed the absolute Pearson correlation coefficients (|r|) between the original, CR-, SNV-, and FD-processed spectra (400–900 nm) and each biophysical trait, with the comparative results summarized in **Figure 5**. For original reflectance, the spectral–trait correlation structure showed a more pronounced shift between the simulated and observed domains across multiple traits, with reduced correlation strength and poorer agreement in the locations of spectrally sensitive regions.

However, for the CR, SNV, and FD-processed spectra, the correlation patterns between simulations and observations show consistency across multiple traits overall. Specifically, for CHL (**Figures 5a, b**), simulated and measured spectra showed highly similar wavelength-dependent correlation profiles. For example, SNV spectra in both datasets displayed pronounced |r| peaks in the green-peak and red-edge regions, indicating stable spectral sensitivity to chlorophyll variability in these bands. For leaf nitrogen content (N, **Figures 5c, d**), simulated spectra displayed relatively low correlations with N, whereas measured spectra showed stronger associations. Moreover, the spectral–trait pattern for N resembled that for chlorophyll. For DLD_CBC_ in the simulations and DLD in the field measurements (**Figures 5e, f**), simulated spectra exhibited slightly higher correlations compared to the measured data, yet the overall fluctuation trends of the correlation curves remained similar.

### 3.3 Boruta-based feature selection for biophysical traits

**Figure 6:**
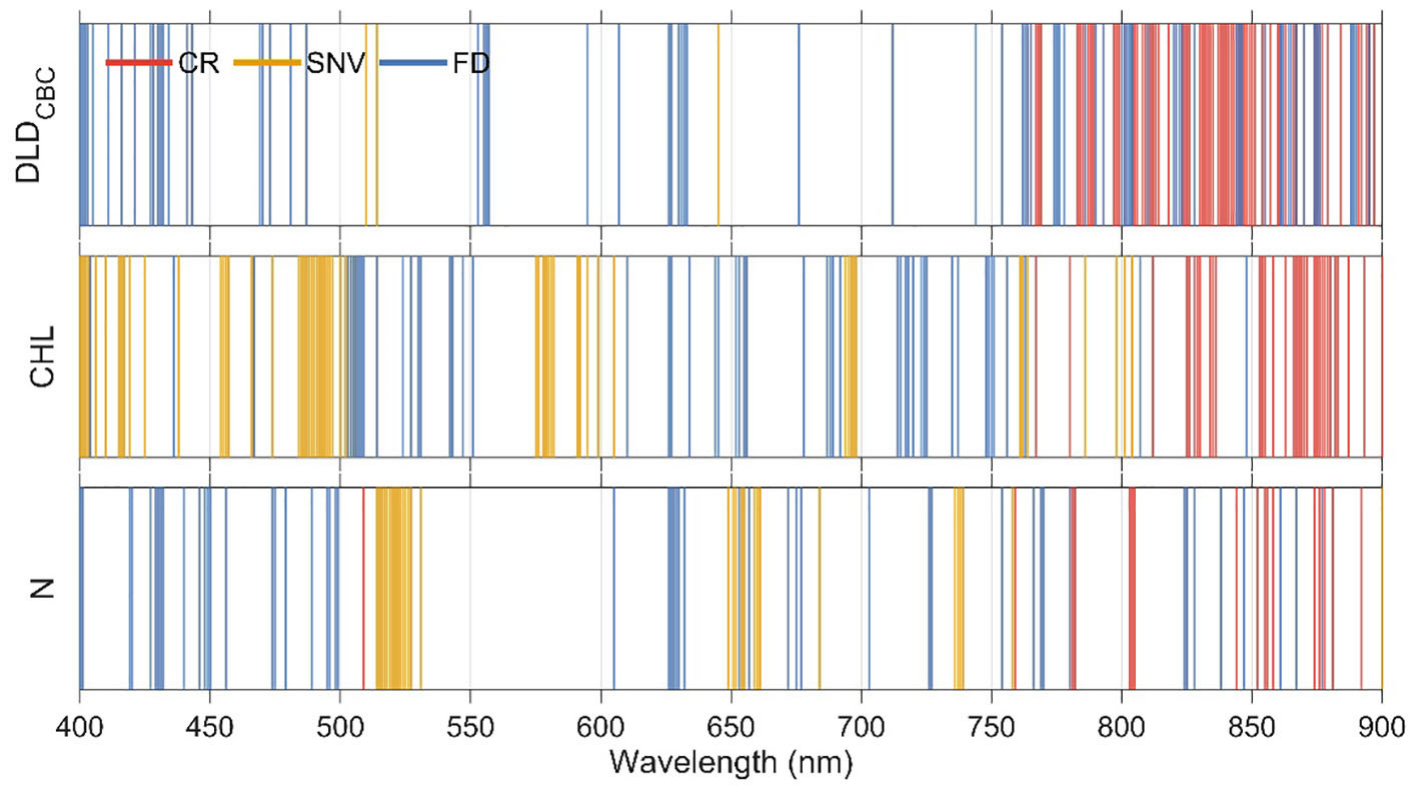
Boruta-based feature selection results for CR-, SNV-, and FD-transformed spectra associated with biophysical traits of *H. hainanensis*.

Due to the limited separability of the original reflectance, all subsequent analyses were performed using scale-standardized spectra only. Integrating CR, SNV, and FD spectra as inputs, the Boruta algorithm was employed to select features targeting each biophysical trait. For CHL, N, and DLD_CBC_ (corresponding to the field-measured DLD), 150, 100, and 150 informative features were selected, respectively (**Figure 6**). Feature selection was performed on the training set. Given the potential domain discrepancy between RTM-simulated and field-measured spectra, we fixed the number of selected features for each trait to ensure sufficient coverage of trait-relevant spectral information in both datasets. Owing to the relatively low correlation between simulated spectra and N compared to the stronger association observed in measured spectra, we retained only 100 features for N to limit the inclusion of low-informative bands that could introduce noise and redundancy. **Figure 6** further indicates that, for chlorophyll and N, all three transformations (CR, SNV, and FD) contributed informative bands across distinct spectral regions: selected chlorophyll-related bands were distributed broadly from the visible to the near-infrared (NIR), whereas N-related bands were concentrated mainly around the green-peak and NIR regions. In contrast, for the more structurally oriented trait DLD_CBC_, CR and FD, transformations that more explicitly emphasize absorption features and spectral-shape changes, provided more informative signals than SNV, with selected features predominantly clustered in the NIR domain.

### 3.4 Inversion of biophysical traits using hybrid models

**Figure 7:**
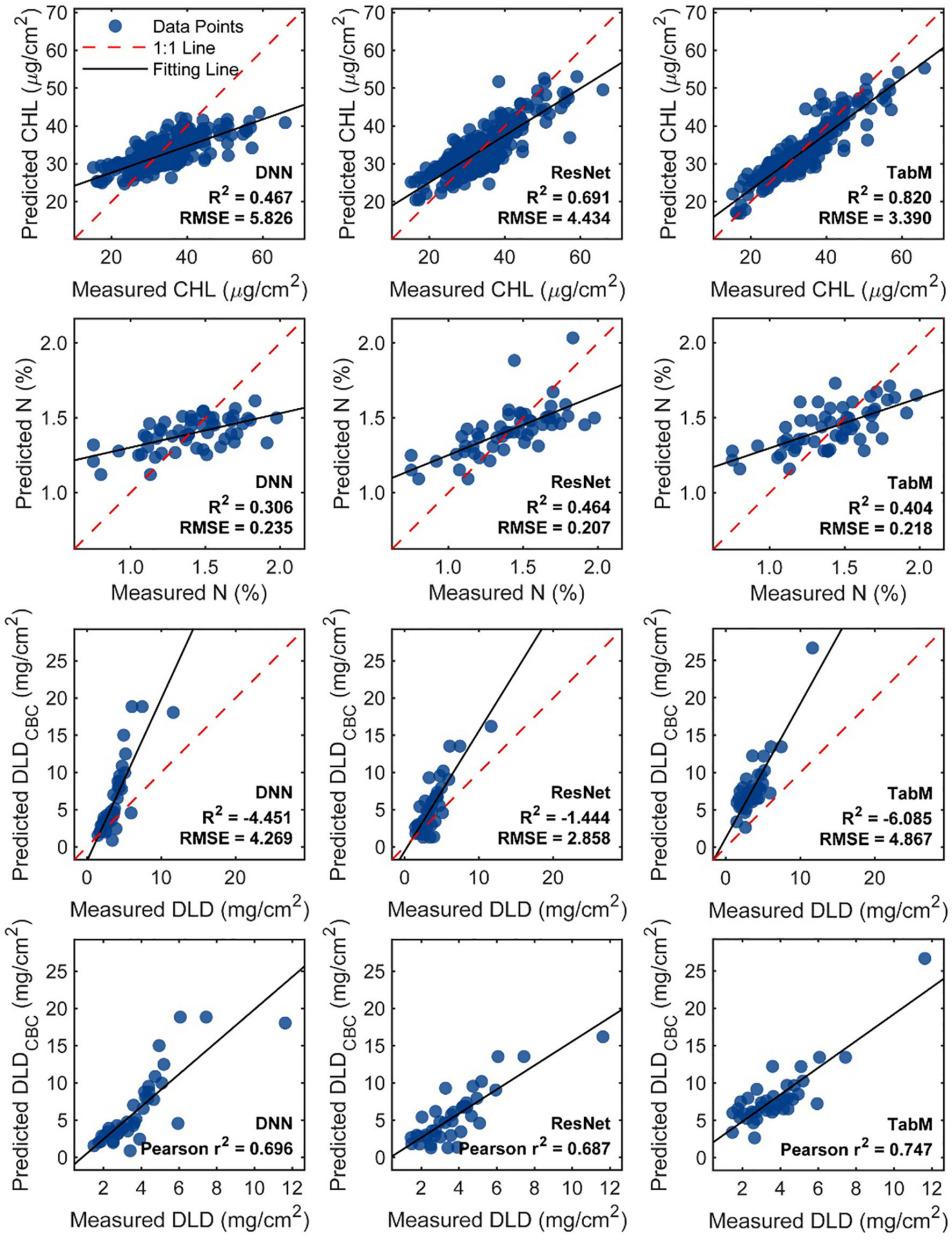
Comparison of test-set performance and predicted–observed relationships for multiple biophysical traits using DNN, ResNet, and TabM models.

Based on the key spectral bands selected by Boruta, this study constructed feature input sets and developed spectral–trait prediction models using simulated data generated by PROSAIL-PRO. Model training and initial validation were conducted using three deep learning architectures (DNN, ResNet, and TabM). The trained models were then applied to field-measured spectra to retrieve biophysical traits, and performance was evaluated against the corresponding ground measurements using standard accuracy metrics (**Table 4**). Validation and test results are summarized in **Table 4**. The agreement between predicted and observed values of the test set, along with their distributional characteristics, is illustrated in **Figure 7**.

**Table 4:** Comparison of validation and test performance of DNN, ResNet, and TabM models for different biophysical traits.

Traits	Model	Validation set		Test set		
		R^2^	RMSE	R^2^	RMSE	Pearson r^2^
CHL (μg·cm^-2^)	DNN	0.941	3.888	0.467	5.826	0.564
	ResNet	0.970	2.778	0.691	4.434	0.700
	TabM	0.994	1.277	**0.820**	3.390	0.833
N	DNN	0.301	3.355	0.306	0.235	0.351
	ResNet	0.313	3.326	**0.464**	0.207	0.477
	TabM	0.553	2.684	0.404	0.218	0.440
DLD_CBC_/DLD (mg·cm^-2^)	DNN	0.882	2.409	-4.451	4.269	0.696
	ResNet	0.914	2.053	-1.444	2.858	0.687
	TabM	0.985	0.874	-6.085	4.867	**0.747**

Bold values indicate the best test-set performance for each trait based on R² for CHL and N and Pearson r² for DLDCBC/DLD.

Validation results on the simulated dataset indicate that all three models achieved high accuracy for CHL and DLD_CBC_. TabM delivered the best overall performance, with validation R^2^ values of 0.994 for CHL and 0.985 for DLD_CBC_, together with the lowest error (RMSE = 1.277 μg·cm^-2^ and 0.874 mg·cm^-2^), followed by the ResNet model. However, for N, the accuracy of all models was markedly lower than for the other traits, and only TabM reached validation R^2^ above 0.5. When evaluated on field-measured spectra, TabM remained the best-performing model for CHL retrieval, achieving a test R^2^ of 0.820 and an RMSE of 3.390 µg·cm^-2^. For N, ResNet showed the strongest performance among the three models, although the test R^2^ remained below 0.5. When using field-measured DLD to validate simulated DLD_CBC_, the resulting R² values were negative, indicating that the one-to-one prediction error was larger than that of a mean-value baseline. Therefore, DLD_CBC_ should not be interpreted as an accurate absolute prediction of DLD on the original numerical scale, because it is a CBC- and LAI-derived canopy proxy rather than a direct equivalent of measured DLD in definition and physical units (Equation 4). We therefore used the squared Pearson correlation coefficient (r²) to evaluate their linear consistency, with TabM showing the strongest agreement (r² = 0.747). This indicates that DLD_CBC_ can reflect relative changes in DLD and provide a proxy indicator of canopy dry-matter status under VNIR-only conditions.

**Figure 7** presents scatter plots of test-set predictions from the DNN, ResNet, and TabM models for different biophysical traits (predicted vs. measured). For CHL, TabM shows the best agreement with observations, with points clustering more tightly around the 1:1 line and exhibiting reduced dispersion. For N, all three models display pronounced systematic bias, characterized by overestimation at low values and underestimation at high values. When model outputs of DLD_CBC_ were directly compared with field-measured DLD (**Figures 7g–i**), predictions are clearly shifted above the 1:1 line, indicating substantial overestimation. This offset suggests that DLD_CBC_ is expressed on a larger numerical scale than DLD. When the 1:1 reference is omitted (**Figures 7j–l**), DLD_CBC_ and DLD nevertheless exhibit a clear linear relationship, suggesting that the models capture coherent directional variation between the two variables.

### 3.5 Response of measured and inverted biophysical traits to shading gradients

**Figure 8:**
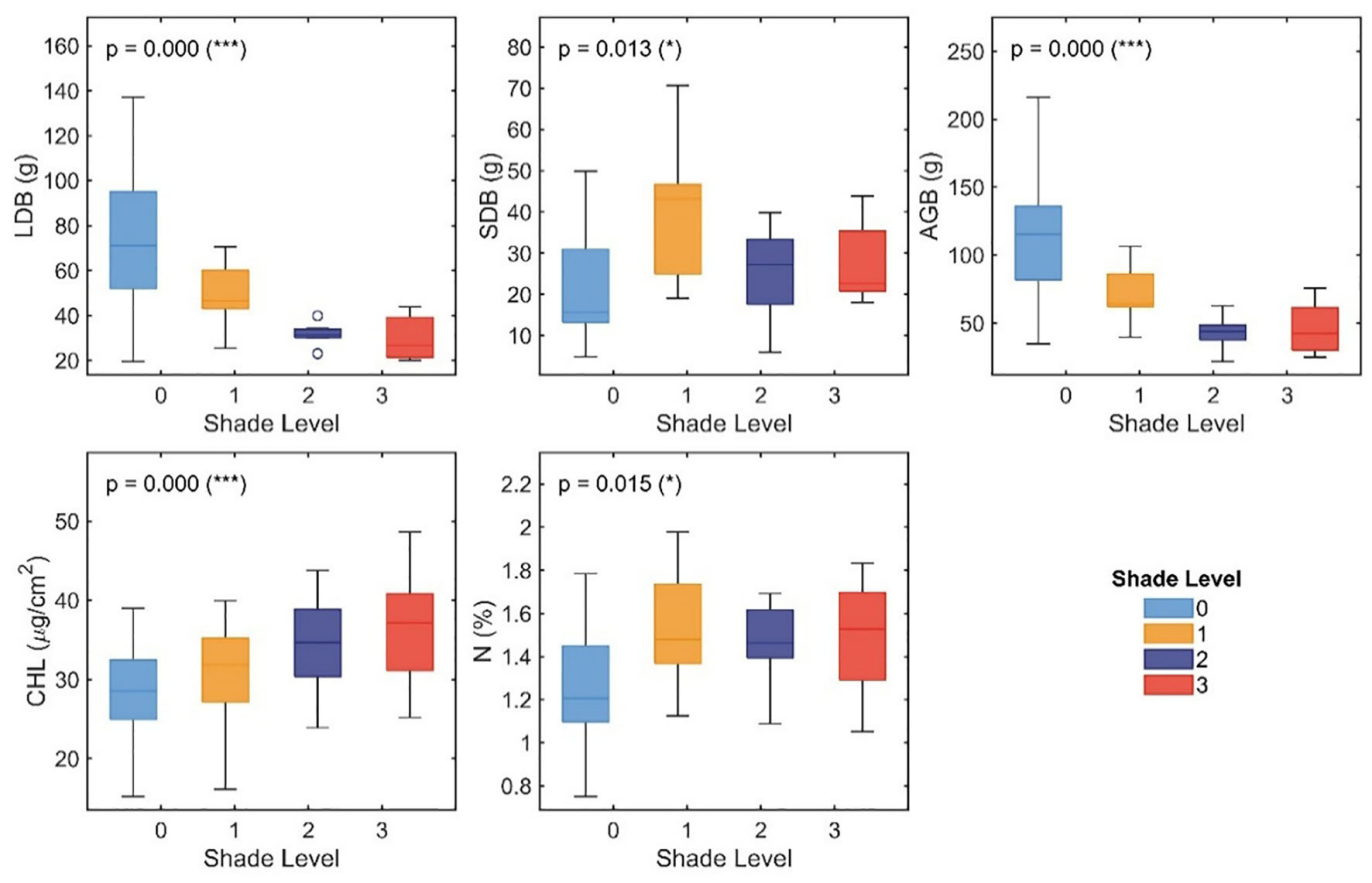
Shading-induced changes in field-measured biophysical traits of *H. hainanensis* and their statistical significance. Asterisks indicate statistical significance among shading treatments: *p < 0.05, **p < 0.01, and ***p < 0.001; ns indicates no statistically significant difference.

**Figure 9:**
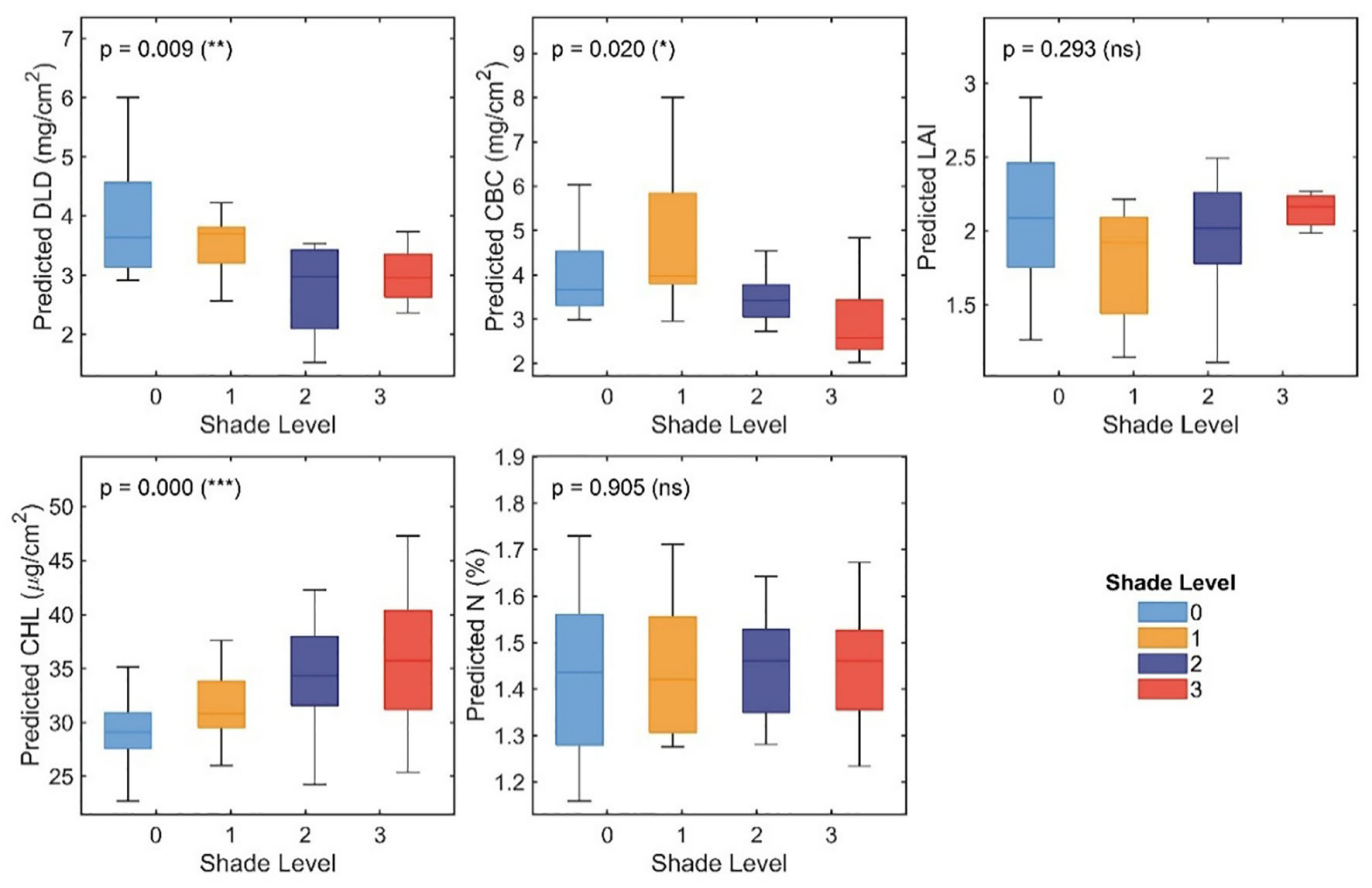
Shading-induced changes in RTM–TabM–retrieved biophysical traits of *H. hainanensis* and their statistical significance. Asterisks indicate statistical significance among shading treatments: *p < 0.05, **p < 0.01, and ***p < 0.001; ns indicates no statistically significant difference.

Trait retrieval results indicate that the RTM–TabM hybrid model can effectively retrieve multiple biophysical traits of *H. hainanensis*. To further examine how observed and retrieved traits respond to shading and whether their response patterns are consistent, we stratified all traits by shading level and summarized group statistics. In the field-measured dataset, results for LDB, SDB, AGB, CHL, and N are presented, whereas in the hybrid-retrieval dataset, results for DLD (obtained by linearly rescaling DLD_CBC_to the DLD scale), CBC, LAI, CHL, and N are reported (**Figures 8**, **9**).

In the field measurements, LDB, SDB, AGB, CHL, and N all showed significant differences across shading levels: LDB and AGB decreased with increasing shade, while CHL and N increased; SDB peaked under light shading (S2). In contrast, for RTM–TabM retrievals, only DLD, CBC, and CHL showed significant differences among shading treatments, whereas LAI and N did not. Moreover, retrieved CHL mirrored the shading response of measured CHL, retrieved DLD exhibited the same directional pattern as measured LDB and AGB, and retrieved CBC, similar to measured SDB, peaked under light shading (S2).

## 4 Discussion

### 4.1 Scale normalization improves spectral instability under high environmental variability caused by shading

Under a shading gradient, the light environment experienced by young *Hopea hainanensis* canopies exhibits significant spatial and temporal heterogeneity (e.g., rapid alternation between sunspots and shade, varying ratios of direct and diffuse light, and dynamic fluctuations in incident and observation angles). This high environmental variability is directly reflected in the raw hyperspectral reflectance of the canopy, manifesting as substantial intragroup fluctuations and instability (Anderson et al., 2011; Pacheco-Labrador and Martín, 2015; Köppl et al., 2021). Although radiation calibration is performed using a standard white panel for each measurement, white panel calibration primarily eliminates scale differences between the instrument response and instantaneous irradiance. It struggles to fully compensate for variations caused by bidirectional reflectance distribution functions (BRDF), shadow proportions, leaf orientation distributions, and background contributions. The results of this study indicate that spectral differences between different shading levels in raw reflectance are easily obscured by intragroup noise across multiple key wavelength bands, particularly in the green peak and red edge regions (**Table 3**, **Figure 5**), thereby validating this perspective. This has direct implications for nondestructive monitoring under shading management. If raw reflectance is used without appropriate normalization, variations caused by illumination geometry and shade intensity may be mixed with trait-related spectral signals, making it difficult to distinguish true physiological or structural changes from measurement-induced spectral variability. Therefore, shading not only modifies sapling growth conditions but also increases the uncertainty of hyperspectral trait retrieval.

In contrast, applying scale-normalization approaches such as CR, SNV and FD, markedly improves the signal-to-noise ratio in the green-peak and red-edge regions (**Table 3**). These findings indicate that scale normalization is not merely a routine preprocessing step, but rather a crucial approach for recovering spectral shape and relative variation information under shaded conditions with high environmental variability. Previous studies have also suggested that mitigating brightness and background effects, while emphasizing absorption features or spectral slopes, can improve the stability and comparability of hyperspectral data (Escribano et al., 2010; Kong et al., 2025). The results of this study further demonstrate that, in shading gradient experiments, scale normalization establishes an essential data foundation for subsequent analysis and cross-domain modeling. Specifically, CR, SNV, and FD reduced illumination-related amplitude variation and enhanced absorption- and shape-related spectral information, thereby improving the reliability of spectral inputs before feature selection and hybrid inversion.

### 4.2 Development and validation of the hybrid retrieval framework for biophysical traits

In this study, we developed and evaluated a hybrid modeling framework integrating RTM and deep learning (DL) to retrieve key biophysical traits of *H. hainanensis* saplings and capture their responses to shading. This approach aligns with recent research trends in hyperspectral inversion, where incorporating physical models to constrain spectral–trait relationships can enhance the robustness and transferability of data-driven models under limited sample conditions (Liu et al., 2025; Yang et al., 2025).

By comparing spectral–trait relationships derived from RTM simulations and field measurements, we found that multiple traits exhibited consistent spectral sensitivity regions across the two domains (**Figure 6**). Similar cross-domain consistency has been reported and is widely regarded as a prerequisite for successful transfer in hybrid modeling (Bhadra et al., 2024). Importantly, our results further suggest that even under a shading gradient that substantially alters incident radiation and adds observational complexity, RTM can still reproduce the core characteristics of the spectral–trait relationships for *H. hainanensis* saplings. This provides a physically grounded basis for pre-training models in the simulated domain and transferring them to field conditions.

From a model-architecture perspective, validation on simulated data consistently indicated that RTM-derived spectral–trait mappings are highly learnable across three deep learning models (DNN, ResNet, and TabM). When deployed on field-measured spectra, TabM, designed under a parameter-efficient ensemble paradigm, exhibited the strongest generalization on the test set, achieving the highest accuracy for chlorophyll retrieval (R^2^ = 0.820; **Figures 7a–c**). This observation is consistent with previous studies highlighting the superior robustness and anti−overfitting capability of ensemble methods (Jiang et al., 2025; Gorishniy et al., 2025), and it further implies that well-designed ensembles may leverage large RTM-generated simulation datasets more effectively, thereby maintaining stable performance under small-sample field settings. The three model architectures provided different levels of nonlinear representation for the selected hyperspectral features. DNN served as a baseline nonlinear model, ResNet introduced residual connections to improve training stability, and TabM used a parameter-efficient ensemble structure to reduce prediction variance. This may explain why TabM showed stronger generalization when transferring from RTM-simulated spectra to field spectra affected by shading-induced variability. However, the limited performance for N indicates that model complexity alone cannot overcome weak VNIR spectral sensitivity; the transferability of trait-specific spectral information remains a key constraint.

At the trait level, the hybrid framework showed clear trait dependence. For traits with well-defined spectral mechanisms and strong cross-domain consistency (e.g., CHL), retrieval performance was robust across model architectures, with TabM showing a particularly pronounced advantage in cross-domain application. In contrast, for traits with weaker direct spectral signals or stronger reliance on covariation (e.g., N), performance remained constrained even with more complex models, reflecting limitations imposed by RTM priors and the size and representativeness of field observations. This pattern is consistent with the long-standing view that N exhibits weak direct spectral signatures and is often retrieved indirectly via correlated biochemical and structural traits (Hallik et al., 2009; Liu et al., 2020).

Overall, these findings demonstrate the applicability of physics–data hybrid modeling for trait retrieval and shading-response analysis in *H. hainanensis* saplings, while also indicating that the attainable performance ceiling depends on the completeness of physical priors and the representativeness of observations. Future work could expand field sampling and incorporate multi-angle and multi-scale measurements to better learn complex covariation structures, or adopt structurally richer 3D RTMs to reduce discrepancies between simulated and measured spectra, thereby enabling more generalizable RTM–DL trait retrieval frameworks.

### 4.3 Trait-specific retrieval performance from VNIR spectra and the effectiveness of DLD_CBC_

The hybrid modeling framework exhibited distinct retrieval accuracy for different biophysical traits, a pattern primarily attributable to their inherent spectral properties. CHL was retrieved with high accuracy in both simulated and measured domains and maintained stability during cross−domain application. This indicates that its spectral signal possesses a direct, well−defined, and transferable physical basis within the visible and red−edge regions, a finding consistent with some hyperspectral studies reporting the relatively straightforward retrieval of chlorophyll (Baranoski and Rokne, 2005; Serrano, 2008; Yuan et al., 2022). It also confirms the efficacy of our hybrid framework for core biochemical traits.

In contrast, retrieval accuracy for N was substantially lower. This pattern is not merely a limitation of model capacity, but rather reflects fundamental constraints in spectral physics: diagnostic nitrogen absorption features are primarily located in the shortwave infrared (SWIR) region (Paz-Kagan et al., 2020; Jenal et al., 2021), whereas in the visible–near-infrared domain, N is typically expressed only indirectly through covariation with chlorophyll and structural traits (**Figures 5b, d**). When this covariation is not consistently represented between the simulated and field domains (**Figures 5c, d**), the model fails to learn a stable, transferable mapping.

Against this background, this study addresses the documented challenge for DLD, similar to N, of difficult and unstable retrieval under VNIR conditions (Wang et al., 2011; Féret et al., 2019; Gara et al., 2021). we propose a proxy metric for DLD (DLD_CBC_) constructed by jointly leveraging CBC and LAI. Our results show that although DLD_CBC_ does not perfectly match measured DLD in numerical scale, both exhibit consistent trends under shading gradients (r² = 0.747), demonstrating that the index can reliably capture relative changes in biomass accumulation. This is particularly important for growth monitoring and response analysis, highlighting the feasibility of expanding the application potential of VNIR bands through trait reconstruction rather than direct inversion.

### 4.4 Trait responses to shading management and implications for non-destructive monitoring

From the perspective of shading management and non-destructive monitoring, a central question is whether retrieved traits can faithfully represent the physiological responses of *H. hainanensis* saplings to shading stress, thereby providing actionable value for management. To address this, we jointly examined shading-induced patterns in field-measured traits and in traits retrieved by the hybrid model, providing direct evidence for the ecological plausibility of model outputs.

Our results reveal clear, physiologically meaningful trait responses along the shading gradient. With increasing shading intensity, DLD and AGB decreased overall, whereas CHL and N increased. This pattern reflects a typical low-light adaptation strategy, consistent with previous studies (Ashton et al., 1999; Sack and Grubb, 2002; Xie et al., 2018), whereby saplings increase areal investment in photosynthetic pigments and N allocation to enhance light-use efficiency, while overall growth remains constrained by light limitation. Notably, SDB peaked under light shading, suggesting that moderate shading may be the optimal choice for stem growth, a phenomenon previously observed in other tree species (Schall et al., 2012; Zhang et al., 2022; Chen et al., 2025). These observed responses provide a reference baseline against which the ecological consistency of retrieved traits can be evaluated. The hybrid model successfully reproduced these shading-response patterns for several key traits. Retrieved CHL increased with shading, closely matching the field observations. In addition, retrieved structure-related indicators (e.g., DLD and CBC) exhibited shading-dependent trends consistent with measured DLD and AGB, including a peak under light shading. This indicates that, despite remaining uncertainties in absolute quantification, the model can reliably capture the direction of physiologically meaningful changes driven by shading gradients, supporting the practical feasibility of hyperspectral, non-destructive monitoring.

Based on these findings, we propose an application-oriented framework for non-destructive monitoring and shading management in *H. hainanensis* saplings: using relative changes in chlorophyll and structure-related traits as core indicators, coupled with hyperspectral hybrid modeling to enable rapid and continuous assessment of shading responses. This framework provides a quantitative pathway for precision cultivation and long-term monitoring of endangered tree saplings.

## 5 Conclusions

This study focused on *H. hainanensis* saplings to develop nondestructive VNIR hyperspectral approaches for monitoring key biophysical traits along a shading gradient. To address the high variability of light environment under shading gradient conditions, we systematically analyzed the applicability and limitations of VNIR canopy hyperspectral data for biophysical trait retrieval. Our results show that the raw reflectance, affected by shading-induced amplitude variation and background effects, failed to stably express differences among shading levels, thereby weakening the physical consistency of the spectral–trait relationship. By integrating scale normalization methods such as CR, SNV, and FD into a hybrid RTM-DL modeling process, this study effectively suppressed spectral noise caused by shading and mitigated the domain shift between simulated and measured data, providing a necessary foundation for robust VNIR-based modeling. Within this framework, CHL exhibited the most stable and reliable cross-domain retrieval performance (R² = 0.820; RMSE = 3.390 μg·cm^-2^), whereas N retrieval remained constrained by the limited physical sensitivity of the VNIR spectral region (R² < 0.5). Moreover, to overcome difficulty in stably retrieving conventional LMA and DLD from VNIR spectra, we proposed a CBC-based proxy metric, DLD_CBC_. It showed strong trend consistency with DLD observations (r² = 0.747; RMSE = 4.867 mg·cm^-2^) and reproducibly captured the directional trait response across the shading gradient, supporting its use as an effective VNIR-compatible proxy for DLD. In summary, this study demonstrates that under high environmental variability from shading, a combined approach of scale normalization, RTM–deep learning hybrid modeling, and VNIR-adapted trait reconstruction enables robust and non-destructive retrieval of key biophysical traits in *H. hainanensis* saplings, offering a practical technical pathway for growth monitoring and precision shading management in endangered tree species.

## Data Availability

The original contributions presented in the study are included in the article/supplementary material. Further inquiries can be directed to the corresponding author.
